# Time‐Variant Patterns of Comorbid Depression and Anxiety Across Early to Late Adolescence: The Sex‐Specific Role of Emotion Regulation

**DOI:** 10.1155/da/7875846

**Published:** 2026-10-29

**Authors:** Dongyu Liu, Xiyu Wei, Luxia Jia, Bao Zhang

**Affiliations:** ^1^ School of Education Guangzhou University Guangzhou China gzhu.edu.cn; ^2^ School of Public Health LKS Faculty of Medicine The University of Hong Kong Hong Kong Special Administrative Region China hku.hk; ^3^ Department of Social Work and Social Administration The University of Hong Kong Hong Kong Special Administrative Region China hku.hk

**Keywords:** anxiety, comorbidity, depression, emotion regulation, time-varying effect modelling

## Abstract

**Aims:**

Depression–anxiety comorbidity is common in adolescence, yet prevention efforts often overlook the nonlinear course of psychosocial development. This study mapped the dynamic risk of depression–anxiety comorbidity across adolescence and examined whether emotion regulation moderated these associations by grade and sex.

**Methods:**

We utilised time‐varying effect modelling (TVEM) on data of youth from all primary and secondary schools in Chenzhou, China (*n* = 58,622; grades 4–12), to map the dynamic change of comorbid risk and potential moderating role of emotion regulation, with consideration of sex difference. Participants reported depressive and anxiety symptoms through the patient health questionnaire and generalised anxiety disorder questionnaire, respectively. The emotion regulation questionnaire (ERQ) measured two dimensions of emotion regulation, cognitive reappraisal and expression suppression.

**Results:**

Overall comorbid risk increased throughout adolescence. Anxiety (OR = 1.67, 95% CI [1.61, 1.74]) and depressive (OR = 1.68, 95% CI [1.50, 1.89]) symptoms had similar overall comorbid risk, while depression‐related risk became stronger than anxiety‐related risk in late adolescence. Sex‐specific patterns revealed higher comorbid risks in females. A gradual risk progression across grades emerged in males, whereas females showed a steeper increase in depression‐associated comorbidity and an anxiety‐associated peak around grade 8 (OR = 1.74, 95% CI [1.69, 1.80]). The role of emotion regulation is more pronounced in females than males. Cognitive reappraisal had a protective role against anxiety‐related comorbid risk (OR = 0.99, 95% CI [0.99, 0.99]) and a maladaptive role for depression‐related comorbid risk (OR = 1.01, 95% CI [1.01, 1.01]). The maladaptive role of expressive suppression for anxiety‐associated comorbidity peaked during middle school for females and only emerged during early adolescence for males.

**Interpretation:**

Depression–anxiety comorbidity showed distinct grade‐ and sex‐specific risk patterns across adolescence. These findings advocate for precise interventions prioritising anxiety management during primary‐to‐middle school transition and depression prevention during late adolescence. Emotion regulation interventions should be tailored by sex, adolescence stage and existing symptoms.

## 1. Introduction

Depression and anxiety are the most common mental health disorders affecting adolescents worldwide, with prevalence rates of 4.4%–5.5% and 1.4%–3.5%, respectively [1] and are frequently comorbid [2, 3]. Comorbidity indicates the co‐occurrence of distinct disorders in the same individual, while a bidirectional relationship existed as anxiety and depressive symptoms can both increase comorbid risk [4]. Comorbidity is more debilitating than either disorder alone, leading to increased suicidality, poorer prognosis, suboptimal treatment response, lower life satisfaction and more physical health problems [3, 5, 6]. Nevertheless, while reducing comorbidity is a priority for adolescent mental health prevention, existing targeted approaches remain focused on static risk factors and symptom severity [7–9], overlooking the rapidly changing biopsychosocial states in adolescence.

Adolescence represents a critical developmental stage characterised by rapid and multifaceted cognitive, emotional and neurobiological transformations [10], alongside environmental shifts through academic transitions, peer group changes and increasing autonomy demands [11]. Preliminary evidence has indicated that the risk of comorbidity between depression and anxiety disorders may vary across substages of adolescence as higher risks of comorbidity emerged in late compared to early adolescence [6, 12, 13]. Nevertheless, contradictory evidence from other studies indicated no significant age difference in the comorbidity risk [14–16]. While previous studies differ by settings and sample characteristics [2], a pervasive limitation contributing to inconsistent findings could be the persistently reliance on coarse categorisations of age range, such as dichotomising participants into early and late adolescence [6, 12–16]. This approach is particularly problematic, as mental and environmental changes in adolescence do not unfold linearly but rather in episodic or cascading patterns [17–19], ranging from the start of hormonal fluctuations at early adolescence [20], the transition from parent‐dominant social support to the increased importance of the peer network around the middle‐school period [21] to intensifying academic demands and future‐oriented stress in the high‐school period [22]. Moreover, the neurological development during adolescence is not a simple continuous progression, with notable maturation of cortical connectivity patterns around middle‐school period [23] and marked difference in timing of development across brain regions [24, 25]. These dynamic developmental processes potentially indicate a nonlinear risk pattern of comorbidity across adolescence. Similarly, gender disparities likely fluctuate dynamically, as the developmental patterns of psychopathology in adolescents tend to differ across sex [26, 27]. Nevertheless, existing studies predominantly focused on static sex difference [5, 28], neglecting a potential sex‐specific time‐variant patterns of comorbidity risk. For instance, female adolescents tend to have earlier pubertal onset [29] and be more sensitive to peer evaluation and social exclusion compared to their male counterparts [30–32]. These sex‐specific features might lead to additional risks and different risk patterns across adolescence stages. Therefore, a more nuanced approach is essential to precisely delineate year‐by‐year fluctuations in comorbidity patterns that are sensitive to the dynamic impacts from the episodic and rapid biopsychosocial developments during adolescence. This will allow us to pinpoint when sex disparities emerge and identify critical risk windows for precise and personalised prevention and intervention efforts that target stage‐matched risks.

Emotion regulation represents a key transdiagnostic factor and a promising target for preventing the comorbidity between depression and anxiety in adolescents [33–35]. Much like the risk patterns themselves, the capacity for emotion regulation could also fluctuate across adolescence stages and sex. Cognitive reappraisal and expressive suppression are the most extensively studied emotion regulation strategies and serve as central targets in psychological interventions for depression and anxiety [36–38]. Specifically, cognitive reappraisal is typically cultivated as an adaptive skill, whereas expression suppression is often addressed as a maladaptive pattern [36–38]. Nevertheless, one meta‐analysis of intervention studies reported inconsistent evidence on links between emotion regulation and symptom severity [39]. It found no significant associations of cognitive reappraisal or expression suppression with symptom severity of depression or anxiety over treatment in adolescents [39]. Although this meta‐analysis pooled trials with heterogeneous intervention modalities and durations, the conflicting findings may also reflect differences in sample characteristics, including age range and sex compositions. This further necessitates a more nuanced approach to identify the critical period across adolescence for these intervention targets to be more effective.

While previous research predominantly focused on static associations and coarse categorizations of age range, the present study utilised a time‐varying modelling approach to explicitly explore the dynamic pattern of concurrent comorbidity risk across sex and adolescence. This approach allows for the estimation of comorbid risk as a nonlinear function of the adolescence stage rather than as a static average. It could illustrate fine‐grained nonlinear patterns of risk and buffering roles through providing year‐by‐year estimates and assess whether the variations are statistically meaningful. We hypothesised that nonlinear patterns of comorbidity risks associated with depressive and anxiety symptoms would emerge across grades 4–12. Moreover, different patterns of comorbidity risks would emerge across sexes. We further hypothesised that nonlinear patterns would emerge for the moderating roles of cognitive reappraisal and expressive suppression in the associations of comorbidity risks with depressive and anxiety symptoms in both sexes.

## 2. Methods

### 2.1. Procedure and Participants

This cross‐sectional study utilised data from a large‐scale school‐level mental health monitoring survey in Chenzhou, China, encompassing all local schools with a total of 58,999 children and adolescents from primary (*n* = 21,500; 36.4%), middle (*n* = 24,854; 42.1%) and high (including academic and vocational schools; *n* = 12,645; 21.5%) schools. The participant group involved students from grade 4 (age 9–11) to grade 12 (age 17–18). Using grade as an indicator of adolescent stage, the present study could better capture the environmental changes in adolescence due to academic transitions and better inform the critical period for school‐based intervention and prevention. Leveraging data from both academic and vocational schools ensured comprehensive coverage of adolescents in the city. A total of 58,622 participants had complete data on variables of interest and were involved in analysis. All participants or their parents were informed about the content and aims of the study, and informed consent was obtained from all participants. The study received ethical approval from the Ethics Committee of the Institute of the University of Guangzhou (2024080) and followed the Strengthening the Reporting of Observational Studies in Epidemiology (STROBE) reporting guidelines (Table S1).

### 2.2. Measurements

#### 2.2.1. Depression and Anxiety

Depressive symptoms were measured by the Chinese version of the 9‐item patient health questionnaire (PHQ‐9) [40]. Participants self‐reported the frequency they experienced the conditions described by each item in the past 2 weeks through a 4‐point Likert scale from 0 (Never) to 3 (Nearly every day). The total score was calculated as the sum of the nine items, with higher scores indicating worse depressive symptoms. A total score reaching 11 or higher was classified as an indicator of clinically significant depression [41]. PHQ‐9 has been widely applied in various population [42], with excellent internal consistency (*α* = 0.90) in our sample. Strict measurement invariance across grades was supported for PHQ‐9 in our sample (Table S2; *χ* ^2^ (451) = 10928.46, ΔCFI = −0.0061, ΔRMSEA = 0.0075 and ΔSRMR = 0.0049).

Anxiety symptoms were measured by the Chinese version of the 7‐item generalised anxiety disorder (GAD‐7) scale [43]. Participants self‐reported the frequency they experienced the conditions described by each item in the past 2 weeks through a 4‐point Likert scale from 0 (Never) to 3 (Nearly every day). The total score was calculated as the sum of the seven items, with higher scores indicating worse anxiety symptoms. The recommended cut‐off point for clinically significant anxiety was set at 10 [44]. GAD‐7 has demonstrated good psychometric properties in the Chinese population [45], with excellent internal consistency (*α* = 0.92) in our sample. Strict measurement invariance across grades was supported for GAD‐7 in our sample (Table S2; *χ* ^2^ (286) = 14608.40, ΔCFI = −0.0061, ΔRMSEA = 0.0124 and ΔSRMR = 0.0039).

Participants were classified as having comorbid depression and anxiety if they report both clinically significant depression and anxiety. The comorbid depression–anxiety symptom score was calculated through summing *z*‐score standardised PHQ‐9 and GAD‐7 scores to capture the overall burden of depressive and anxiety symptoms [46].

#### 2.2.2. Emotion Regulation

Emotion regulation was measured by the 10‐item emotion regulation questionnaire (ERQ) [47]. It measures two strategies that participants might use to regulate their emotions, including cognitive reappraisal and expressive suppression. Participants self‐reported to what extent they agree with each item on a 7‐point Likert‐type scale ranging from 1 (strongly disagree) to 7 (strongly agree), with higher scores indicating a greater tendency of the participant to use the respective strategy. The ERQ has demonstrated good psychometric properties in Chinese population [48], with excellent internal consistency for cognitive reappraisal (*α* = 0.90) and acceptable internal consistency for expressive suppression (*α* = 0.78) in our sample. Strict measurement invariance across grades was supported for cognitive reappraisal in our sample (Table S2; *χ* ^2^ (265) = 15,064.26, ΔCFI = −0.0060, ΔRMSEA = 0.0122 and ΔSRMR = 0.0047). Scalar measurement invariance across grades was supported for expression suppression in our sample (Table S2; *χ* ^2^ (106) = 1025.86, ΔCFI = −0.0016, ΔRMSEA = −0.0118 and ΔSRMR = −0.0027).

#### 2.2.3. Covariates

Participants indicated their biological sex (male or female) and reported their perceived socio‐economic status based on a subjective 10‐point Likert scale from 1 to 10, with higher scores indicating a higher perceived socio‐economic status.

### 2.3. Statistics

Statistical analyses were conducted through R 4.4.2 [49]. Descriptive statistics were conducted for all variables of interest. Mean, standard deviation, median and range were reported for continuous variables, while percentages were reported for categorical variables.

Time‐varying effect modelling (TVEM) was applied to explore the dynamic associations between depressive and anxiety symptoms and comorbidity from 4th to 12th grades through the *gam* function in the *mgcv* package [50]. TVEM extends regression by allowing coefficients to vary over time and can be applied to cross‐sectional data indexed by time‐like variables, such as age or grade [51, 52]. The models used thin plate regression splines as a smoothing function on the data at discrete time points (grade) to create a curve representing the dynamic trajectory of the associations from 4th to 12th grades [51]. The maximum number of knots were set as nine according to number of grades in our data, while the models penalise the complexity of the curve through restricted maximum likelihood method.

A series of models were conducted in parallel to explore the risk of comorbidity across grades. Sex and perceived socio‐economic status were controlled for in the models as fixed‐effect covariates. Time‐varying associations between depressive and anxiety symptoms were first explored in all participants (*n* = 58,622). Second, the risk of having comorbid depression and anxiety associated with severity of depressive and anxiety symptoms were separately explored in symptomatic participants with either or both clinically significant depression and anxiety (*n* = 20,473) to avoid potential reflection problem [53], where the outcome is a deterministic function of the two exposures, and adolescents below both thresholds cannot be comorbid by definition. Within the symptomatic subgroup, the estimated odds ratios therefore quantify the conditional association between symptom severity in one domain and being in a comorbid rather than a single‐domain clinically significant state. Sensitivity analyses were conducted to have the overall burden of depressive and anxiety symptoms scores as an outcome in all participants. Continuous variables were *Z*‐score standardised before modelling continuous outcomes. Exposure variables were transformed into the same harmonised scale (0–27 for depression and anxiety; 0–30 for emotion regulation variables) before modelling binary comorbidity outcome to ensure meaningful comparison while avoiding overly large uninterpretable estimates. Overall parametric and smooth estimates were reported for each model. In each model, parametric coefficients were also estimated for each grade. Significance were indicated by *p*‐value smaller than 0.05, and 95% confidence interval excluding null estimate (0 and 1 for β and odds ratio, respectively).

To formally compare the magnitude of associations estimated from the parallel models, we conducted a paired nonparametric bootstrap in which both anxiety and depression models were refitted within each bootstrapped dataset (*B* = 1000). In each bootstrap iteration, participants were resampled with replacement, and both parallel TVEMs were refitted using the same resampled dataset. Bootstrap 95% confidence intervals and *p*‐values were calculated for the effect sizes of both overall parametric coefficients and grade‐specific coefficients. For binary outcomes, we calculated the difference between the corresponding log‐ORs for anxiety and depressive symptoms and exponentiated this difference to obtain the ratio of the ORs. For continuous outcomes, we calculated the difference between standardised *βs*.

Sex‐stratified models were also conducted to explore the associated in females and males specifically, with perceived socio‐economic status adjusted for as fixed effect covariate. Then, the potential moderating role of cognitive reappraisal and expression suppression in the associations of comorbidity with depressive and anxiety symptoms were explored in each sex. Stratified analyses were further conducted for participants with high and low scores of cognitive reappraisal and expression suppression. High and low scores were defined as having the score in the top 75% and bottom 25% quantile in the corresponding grade, respectively.

## 3. Results

Among the 58,622 eligible participants (45.9% female), 28.1% reported clinically significant depression, 26.6% reported clinically significant anxiety, while 20.1% reported comorbidity (Table S3). As shown in Figure 1, the overall trend of both individual and comorbid risk of depression and anxiety increased from early to late adolescence, with girls having a stronger difference across grades (Figure S1).

**Figure 1:**
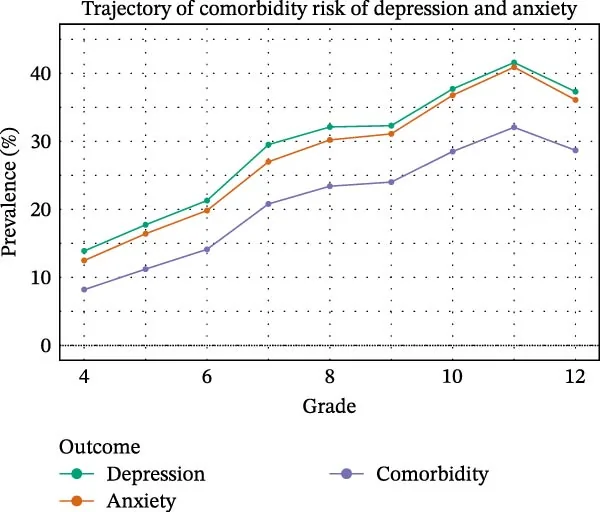
Prevalence of depression and anxiety comorbidity across educational stages.

### 3.1. Comorbidity Between Depression and Anxiety

The time‐varying effect model revealed a significant overall association between depressive and anxiety symptoms (*β* = 0.84, SE = 0.02, *p* < 0.001), while the associations significantly vary by grade (*Edf* = 3.42, *F* = 7.62, *p* < 0.001). As shown in Figure S2, the associations between depression and anxiety symptoms appeared to be strongest in middle adolescents from grade 6 to grade 8, with *β* coefficients vary from 0.76 to 0.83 across grades (Table S4).

In symptomatic participants with either or both clinically significant depression and anxiety, the time‐varying effect model indicated similar overall risks (OR ratio = 0.99, 95% CI [0.96, 1.08]) associated with anxiety symptoms (OR = 1.67, 95% CI [1.61, 1.74]) and depressive symptoms (OR = 1.68, 95% CI [1.50, 1.89]). As shown in Figure 2A, the associations of both anxiety (*Edf* = 3.39, *χ* ^2^ = 20.02, *p* < 0.001) and depressive symptoms (*Edf* = 0.99, *χ* ^2^ = 57.20, *p* < 0.001) significantly increased from grade 4 to grade 12 in symptomatic participants. As shown in Table 1, the comorbidity risk associated with anxiety symptoms showed a notable increase from grade 4 (OR = 1.59, 95% CI [1.51, 1.66]) to grade 8 (OR = 1.68, 95% CI [1.62, 1.72]). The risk associated with depressive symptoms gradually increase by grade in symptomatic participants, as the risk associated with depressive symptoms (OR = 1.87, 95% CI [1.66, 2.10]) became significantly higher than anxiety symptoms (OR = 1.68, 95% CI [1.60, 1.77]) at grade 12 (OR ratio = 0.90, 95% CI [0.86, 0.98]) on the harmonised scale. As shown in Figure S3A, the sensitivity analysis in full sample showed a similar pattern for depression‐related risks (*β* = 1.82, SE = 0.02, *p* < 0.001), which gradually increased with grade (*Edf* = 1.01, *F* = 9.47, *p* < 0.001). A curve pattern for anxiety‐related risks also emerged (*β* = 1.84, SE = 0.02, *p* < 0.001), which coincides with the peak in the transition from primary to middle school (Table S6; *Edf* = 3.42, *F* = 7.61, *p* < 0.001) as identified in the binary model.

**Figure 2:**
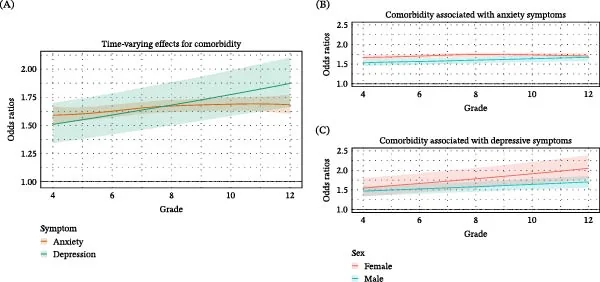
Risk of comorbidity associated with depressive and anxiety symptoms in (A) overall participants and (B, C) by sex. In participants with either or both clinically significant depression and anxiety (*n* = 20,473). Comorbidity was defined as having both clinically significant depression and anxiety. Sex and perceived socioeconomic status were adjusted as fixed‐effect covariates in overall participants; perceived socioeconomic status was adjusted as fixed effect covariate in sex‐stratified models.

**Table 1:** Parametric coefficient of the risk of comorbidity associated with anxiety and depressive symptoms at each grade.

Grade (Age IQR)	Parametric coefficient OR [95% CI]								
	Overall sample			Females			Males		
	Anxiety	Depression	Ratio	Anxiety	Depression	Ratio	Anxiety	Depression	Ratio
G4 (10–11)	1.59 [1.51, 1.66]	1.51 [1.34, 1.70]	1.05 [0.95, 1.08]	1.66 [1.60, 1.73]	1.55 [1.33, 1.81]	1.07 [0.95, 1.10]	1.53 [1.45, 1.62]	1.47 [1.35, 1.61]	1.04 [0.94, 1.11]
G5 (11–12)	1.60 [1.54, 1.66]	1.55 [1.38, 1.74]	1.03 [0.97, 1.07]	1.68 [1.63, 1.73]	1.61 [1.38, 1.87]	1.05 [0.97, 1.08]	1.55 [1.47, 1.63]	1.50 [1.38, 1.63]	1.03 [0.96, 1.09]
G6 (11–13)	1.63 [1.57, 1.68]	1.59 [1.42, 1.79]	1.02 [0.98, 1.06]	1.71 [1.66, 1.76]	1.67 [1.44, 1.93]	1.02 [0.97, 1.07]	1.57 [1.49, 1.65]	1.53 [1.41, 1.66]	1.03 [0.97, 1.07]
G7 (12–14)	1.65 [1.60, 1.71]	1.63 [1.46, 1.83]	1.01 [0.98, 1.05]	1.73 [1.68, 1.79]	1.73 [1.49, 2.00]	1.00 [0.97, 1.08]	1.58 [1.51, 1.66]	1.56 [1.43, 1.69]	1.02 [0.98, 1.06]
G8 (13–14)	1.68 [1.62, 1.72]	1.68 [1.50, 1.88]	0.99 [0.97, 1.04]	1.74 [1.69, 1.80]	1.79 [1.55, 2.07]	0.98 [0.94, 1.04]	1.60 [1.52, 1.68]	1.58 [1.46, 1.72]	1.01 [0.98, 1.06]
G9 (14–15)	1.68 [1.62, 1.73]	1.73 [1.54, 1.93]	0.97 [0.95, 1.02]	1.74 [1.69, 1.80]	1.85 [1.60, 2.14]	0.94 [0.91, 0.99]	1.62 [1.54, 1.70]	1.61 [1.49, 1.75]	1.00 [0.97, 1.06]
G10 (15–16)	1.69 [1.63, 1.75]	1.77 [1.58, 1.99]	0.95 [0.92, 1.00]	1.73 [1.68, 1.79]	1.92 [1.65, 2.22]	0.90 [0.87, 0.96]	1.64 [1.56, 1.72]	1.64 [1.51, 1.79]	1.00 [0.95, 1.08]
G11 (16–17)	1.69 [1.62, 1.76]	1.82 [1.62, 2.05]	0.93 [0.89, 1.00]	1.72 [1.67, 1.78]	1.99 [1.71, 2.31]	0.87 [0.81, 0.93]	1.66 [1.57, 1.75]	1.68 [1.54, 1.82]	0.99 [0.93, 1.10]
G12 (17–18)	1.68 [1.60, 1.77]	1.87 [1.66, 2.10]	0.90 [0.86, 0.98]	1.71 [1.64, 1.78]	2.06 [1.77, 2.39]	0.83 [0.76, 0.90]	1.67 [1.58, 1.77]	1.71 [1.56, 1.86]	0.98 [0.91, 1.13]

*Note:* In participants with either or both clinically significant depression and anxiety (*n* = 20,473); Comorbidity was defined as having both clinically significant depression and anxiety; IOR, Interquartile range; Ratio indicates the difference between anxiety and depression‐related ORs at each grade, with Ratio >1 indicating higher anxiety‐related than depression related OR. Sex and perceived socioeconomic status were adjusted as fixed‐effect covariates in overall participants; Perceived socioeconomic status was adjusted as fixed effect covariate in sex‐stratified models.

### 3.2. Comorbidity Risk by Sex

When having sex as a moderator in symptomatic participants, significant moderating effects of sex emerged for both anxiety (OR = 0.91, 95% CI [0.88, 0.95]) and depressive (OR = 0.89, 95% CI [0.84, 0.94]) symptoms in their associations with comorbidity risk, with significant variations of associations across grades for both interaction terms with anxiety (*Edf* = 2.51, *χ* ^2^ = 17.91, *p* < 0.001) and depressive symptoms (*Edf* = 0.90, *χ* ^2^ = 8.99, *p* = 0.002).

The stratified analysis in symptomatic participants showed that females reported significantly greater risk of comorbidity associated with both anxiety (OR = 1.76, 95% CI [1.69, 1.83]; OR ratio = 0.92, [0.87, 0.97]) and depressive (OR = 1.80, 95% CI [1.55, 2.08]; OR ratio = 0.91, [0.86, 0.98]) symptoms than male participants (anxiety OR = 1.60, 95% CI [1.52, 1.69]; depression OR = 1.59, 95% CI [1.46, 1.72]). As illustrated in Figure 2B,C, while the risks of comorbidity associated with both anxiety (*Edf* = 0.91, *χ* ^2^ = 10.38, *p* < 0.001) and depressive (Edf = 0.95, *χ* ^2^ = 17.23, *p* < 0.001) symptoms gradually increased with grade in males, a relatively sharper increase of the association between depressive symptoms and comorbidity emerged in females (*Edf* = 0.98, *χ* ^2^ = 42.43, *p* = 0.003), as the between sex difference in depression‐related comorbid risk became significant since grade 5 (Table S5). Moreover, as shown in Table S5, the association between anxiety symptoms and comorbidity in females first increased with grade, peaked at grades 8–9, then gradually reduced to become similar to males since grade 11 (Edf = 0.98, *χ* ^2^ = 42.42, *p* < 0.001). As shown in Figure S3B, the sensitivity analysis in full sample also showed small but significant curved patterns in anxiety‐related risk associated with combined symptom severity in both female (*β* = 1.84, SE = 0.03, *p* < 0.001; *Edf* = 3.25, *F* = 6.41, *p* < 0.001) and male participants (*β* = 1.84, SE = 0.02, *p* < 0.001; *Edf* = 2.69, *F* = 1.48, *p* = 0.003; Table S6). A significantly stronger association between anxiety and combined symptoms emerged in females than males in grades 4–9 (Table S7) supporting the difference identified in the binary model. As shown in Figure S3C, the sensitivity analysis in full sample showed a gradual increase in the association between depressive symptoms across grade in both female (*β* = 1.82, SE = 0.02, *p* < 0.001; *Edf* = 1.82, *F* = 4.33, *p* < 0.001) and male participants (*β* = 1.80, SE = 0.02, *p* < 0.001; *Edf* = 1.01, *F* = 4.44, *p* < 0.001). A significantly stronger association between depressive and combined symptoms emerged in females than males across grades (Table S7), supporting the difference identified in the binary model.

### 3.3. Sex‐Specific Moderating Role of Emotion Regulation

As shown in Figure S4 and Table S8, while expression suppression emerged as a grade‐nonvariant risk factor in symptomatic participants for comorbidity between depression and anxiety (OR = 1.04, 95% CI [1.04, 1.05]), the protective role of cognitive reappraisal (OR = 0.94, 95% CI [0.92, 0.97]) gradually increased with grade (*Edf* = 0.97, *χ* ^2^ = 21.97, *p* < 0.001).

In symptomatic participants, significant grade–nonvariant interaction of cognitive reappraisal emerged in females with both anxiety (OR = 0.99, 95% CI [0.99, 0.99]) and depressive symptoms (OR = 1.01, 95% CI [1.01, 1.01]) in the association with comorbidity. No significant interaction emerged for cognitive reappraisal in males with either anxiety (OR = 1.00, 95% CI [1.00, 1.00]) or depression (OR = 1.00, 95% CI [1.00, 1.00]). As for expression suppression, grade–variant interaction in females emerged with anxiety symptoms (OR = 1.01, 95% CI [1.01, 1.02]; *Edf* = 3.12, *χ* ^2^ = 14.74, *p* < 0.001), which peaked at grade 8 (Table S9). No significant interaction emerged for females between expression suppression and depression (OR = 1.00, 95% CI [0.99, 1.00]). In males, no overall significant interaction emerged for expression suppression with either anxiety (OR = 1.00, 95% CI [1.00, 1.01]) or depression (OR = 1.00, 95% CI [1.00, 1.01]). Grade–variant interaction for males emerged between expression suppression and depressive symptoms (*Edf* = 0.88, *χ* ^2^ = 7.48, *p* = 0.001), which became significant after grade 9 (Table S9).

The stratified analysis in females (Figure 3A–D) showed stronger associations between anxiety symptoms and comorbidity (OR = 0.91, 95% CI [0.79, 0.99]) in low cognitive reappraisal group (OR = 1.83, 95% CI [1.73, 1.94]) compared to high cognitive reappraisal group (OR = 1.66, 95% CI [1.57, 1.75]). Conversely, no significant overall difference (OR = 1.09, 95% CI [0.96, 1.25]) emerged between depressive symptoms and comorbidity in high cognitive reappraisal group (OR = 1.89, 95% CI [1.78, 2.02]) and low cognitive reappraisal group (OR = 1.73, 95% CI [1.46, 2.05]). Nevertheless, grade‐specific difference existed, as the high cognitive reappraisal group showed significantly higher depression‐related comorbid risk from grade 4 to grade 9 (Table 2). A significantly stronger overall association (OR = 1.17, 95% CI [1.09 1.46]) also emerged between anxiety symptoms and comorbidity in high expression suppression group (OR = 1.89, 95% CI [1.76, 2.02]) compared to low expression suppression group (OR = 1.61, 95% CI [1.54, 1.68]). Moreover, a curve of female‐specific associations across grade only emerged in the high expression suppression group, with the association between anxiety symptoms and comorbidity peaked at grade 8 (Table 2). The stratified analysis in males (Figure 4A–D) showed weaker associations between anxiety symptoms and comorbidity before grade 5, and stronger associations between depressive symptoms and comorbidity in the low expression suppression group after grade 10 (Table 2).

**Figure 3:**
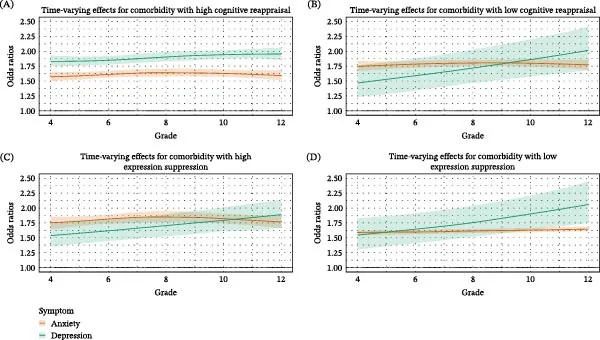
Risk of comorbidity associated with anxiety and depressive symptoms by (A, B) cognitive reappraisal and (C, D) expression suppression in female adolescents. In participants with either or both clinically significant depression and anxiety. Comorbidity was defined as having both clinically significant depression and anxiety. Sex and perceived socioeconomic status were adjusted as fixed‐effect covariates. High and low scores were defined as having the score in the top 75% and 25% quantile in the corresponding grade, respectively.

**Figure 4:**
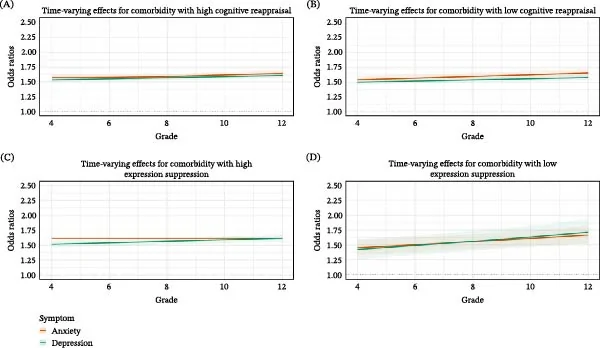
Risk of comorbidity associated with anxiety and depressive symptoms by (A, B) cognitive reappraisal and (C, D) expression suppression in male adolescents. In participants with either or both clinically significant depression and anxiety. Comorbidity was defined as having both clinically significant depression and anxiety. Sex and perceived socioeconomic status were adjusted as fixed‐effect covariates. High and low scores were de fined as having the score in the top 75% and 25% quantile in the corresponding grade, respectively.

**Table 2:** Parametric coefficients of the risk of comorbidity associated with anxiety and depressive symptoms at each grade by emotion regulation score in female and male adolescents.

Grade	Parametric coefficient OR [95% CI]							
	Female adolescents				Male adolescents			
	Anxiety symptoms		Depressive symptoms		Anxiety symptoms		Depressive symptoms	
	High score	Low score	High score	Low score	High score	Low score	High score	Low score
Stratified by cognitive reappraisal								
G4	1.57 [1.49, 1.65]	1.75 [1.67, 1.83] ^∗^	1.83 [1.73, 1.93]	1.47 [1.22, 1.76] ^∗^	1.58 [1.52, 1.64]	1.54 [1.48, 1.61]	1.54 [1.50, 1.58]	1.50 [1.46, 1.55]
G5	1.59 [1.52, 1.65]	1.76 [1.70, 1.83] ^∗^	1.83 [1.75, 1.91]	1.53 [1.28, 1.82] ^∗^	1.58 [1.53, 1.63]	1.56 [1.50, 1.62]	1.55 [1.51, 1.59]	1.51 [1.47, 1.56]
G6	1.61 [1.55, 1.67]	1.78 [1.72, 1.84] ^∗^	1.85 [1.78, 1.92]	1.59 [1.34, 1.89] ^∗^	1.58 [1.54, 1.63]	1.57 [1.51, 1.63]	1.56 [1.52, 1.60]	1.52 [1.48, 1.57]
G7	1.63 [1.57, 1.70]	1.80 [1.74, 1.85] ^∗^	1.88 [1.80, 1.95]	1.65 [1.40, 1.95] ^∗^	1.59 [1.54, 1.63]	1.58 [1.52, 1.65]	1.57 [1.53, 1.61]	1.53 [1.49, 1.58]
G8	1.64 [1.58, 1.71]	1.80 [1.75, 1.86] ^∗^	1.90 [1.83, 1.98]	1.72 [1.46, 2.03] ^∗^	1.60 [1.55, 1.64]	1.60 [1.54, 1.66]	1.57 [1.54, 1.61]	1.54 [1.50, 1.58]
G9	1.64 [1.57, 1.70]	1.80 [1.75, 1.86] ^∗^	1.93 [1.85, 2.01]	1.79 [1.52, 2.11] ^∗^	1.61 [1.56, 1.65]	1.61 [1.55, 1.68]	1.58 [1.54, 1.62]	1.55 [1.51, 1.60]
G10	1.63 [1.57, 1.69]	1.80 [1.74, 1.86] ^∗^	1.94 [1.87, 2.02]	1.86 [1.57, 2.20]	1.62 [1.58, 1.67]	1.63 [1.56, 1.69]	1.59 [1.55, 1.63]	1.56 [1.52, 1.61]
G11	1.61 [1.54, 1.68]	1.79 [1.72, 1.86] ^∗^	1.95 [1.87, 2.04]	1.94 [1.63, 2.31]	1.63 [1.58, 1.69]	1.64 [1.58, 1.71]	1.60 [1.56, 1.65]	1.57 [1.53, 1.62]
G12	1.59 [1.51, 1.68]	1.77 [1.69, 1.86] ^∗^	1.95 [1.85, 2.06]	2.02 [1.68, 2.42]	1.65 [1.58, 1.71]	1.66 [1.59, 1.73]	1.61 [1.57, 1.66]	1.58 [1.53, 1.63]
Stratified by expression suppression								
G4	1.74 [1.64, 1.86]	1.58 [1.54, 1.62]	1.53 [1.35, 1.75]	1.54 [1.30, 1.82]	1.61 [1.61, 1.62]	1.45 [1.33, 1.58] ^∗^	1.52 [1.46, 1.57]	1.42 [1.26, 1.60]
G5	1.77 [1.69, 1.87]	1.59 [1.55, 1.62] ^∗^	1.57 [1.39, 1.78]	1.59 [1.35, 1.86]	1.61 [1.61, 1.62]	1.48 [1.36, 1.61] ^∗^	1.53 [1.47, 1.58]	1.46 [1.30, 1.63]
G6	1.81 [1.72, 1.90]	1.59 [1.56, 1.63] ^∗^	1.62 [1.44, 1.82]	1.64 [1.40, 1.91]	1.61 [1.61, 1.62]	1.50 [1.39, 1.63] ^∗^	1.54 [1.49, 1.59]	1.49 [1.34, 1.66]
G7	1.84 [1.75, 1.93]	1.60 [1.57, 1.64] ^∗^	1.66 [1.48, 1.86]	1.69 [1.45, 1.97]	1.61 [1.61, 1.62]	1.53 [1.41, 1.65] ^∗^	1.55 [1.50, 1.61]	1.53 [1.37, 1.70]
G8	1.85 [1.76, 1.94]	1.61 [1.57, 1.64] ^∗^	1.70 [1.52, 1.90]	1.76 [1.51, 2.04]	1.61 [1.61, 1.62]	1.56 [1.44, 1.68]	1.57 [1.51, 1.62]	1.56 [1.41, 1.74]
G9	1.84 [1.75, 1.93]	1.62 [1.58, 1.65] ^∗^	1.74 [1.56, 1.95]	1.82 [1.57, 2.12]	1.61 [1.61, 1.62]	1.58 [1.46, 1.71]	1.58 [1.52, 1.63]	1.60 [1.44, 1.78]
G10	1.82 [1.73, 1.91]	1.63 [1.59, 1.66] ^∗^	1.79 [1.59, 2.01]	1.89 [1.62, 2.21]	1.61 [1.61, 1.62]	1.61 [1.49, 1.75]	1.59 [1.54, 1.65]	1.64 [1.47, 1.82]
G11	1.79 [1.70, 1.88]	1.63 [1.60, 1.67] ^∗^	1.84 [1.63, 2.07]	1.98 [1.68, 2.32]	1.61 [1.61, 1.62]	1.64 [1.51, 1.78]	1.60 [1.55, 1.66]	1.68 [1.50, 1.88] ^∗^
G12	1.76 [1.65, 1.87]	1.64 [1.60, 1.68] ^∗^	1.88 [1.66, 2.14]	2.06 [1.73, 2.43]	1.61 [1.61, 1.62]	1.67 [1.53, 1.82]	1.61 [1.56, 1.67]	1.71 [1.52, 1.93] ^∗^

*Note:* In participants with either or both clinically significant depression and anxiety. Comorbidity was defined as having both clinically significant depression and anxiety. Sex and perceived socioeconomic status were adjusted as fixed‐effect covariates. High and low scores were defined as having the score in the top 75% and 25% quantile in the corresponding grade, respectively.

^∗^Significant difference in ORs between participants with high and low scores at *p* < 0.05.

## 4. Discussion

By leveraging a large‐scale dataset and TVEM, this study delineates the nonlinear pattern of depression–anxiety comorbidity from early to late adolescence. Our analysis revealed that the risk of comorbidity is dynamically associated with the developmental stage and biological sex, challenging static models of adolescent psychopathology. We observed three principal patterns. First, anxiety and depressive symptoms showed broadly comparable overall associations with comorbidity risk, which escalates from grades 4 to 12. Grade‐varying estimates indicated anxiety symptoms‐related comorbid risk only escalated before grade 8, while depressive symptoms‐related comorbid risk continued increasing across grades and became significantly higher than anxiety‐related risk at grade 12. This difference is especially more pronounced in females, in which depression‐associated risk became significantly higher than anxiety‐associated risk since grade 9. Moreover, distinct sex‐specific trajectories were evident, as males exhibited a generally gradual and linear progression in comorbid risk, whereas females displayed a steeper acceleration in depression‐associated comorbidity and a unique peak in anxiety‐associated risk around grade 8. Furthermore, the role of emotion regulation was found to be developmentally contingent in symptomatic participants. The protective benefit of cognitive reappraisal amplified with increasing grade level, suggesting a maturation effect, while the detrimental impact of expressive suppression manifested during specific windows of vulnerability, peaking in middle adolescence for females.

Our findings indicated a progressive escalation in the risk of depression–anxiety comorbidity from early to late adolescence, a trend that aligns with previous evidence suggesting a higher risk of comorbidity in late adolescence [6, 12, 13]. This general increase likely reflected the cumulative burden of psychosocial stressors and biological maturation characteristic of this developmental period [54]. Comparing between anxiety and depression, our results supported a bidirectional association, where both were significantly and comparably associated with comorbidity overall [4]. One interesting result is that depression‐associated comorbid risk escalated more sharply compared to anxiety on harmonised scale and became stronger than anxiety‐associated risk in late adolescence. This may reflect the growing developmental importance of depressive symptoms during late adolescence, when more salient academic, interpersonal and identity‐related stressors are accompanied by marked biological changes [55]. Beyond the aggregate rise in risk, our TVEM identified a critical period of vulnerability during the transition from primary to middle school (grades 6–8), where the association between anxiety symptoms and comorbid risk peaked significantly. This dynamic change supported an episodic and cascading developing patterns in adolescents [17–19], and potentially explained the conflict evidence regarding the age effect on comorbidity from studies dichotomising age into broad categories [14–16]. Moreover, this temporal risk peak may reflect the convergence of biological maturation and school‐contextual changes during early adolescence. Biologically, this period overlaps with the onset of puberty and the remodelling of limbic‐cortical circuits involved in affect regulation, potentially heightening emotional reactivity [10, 56]. Environmentally, the transition to middle school introduces complex social hierarchies and increased academic demands [11]. The heightened sensitivity to peer evaluation and social exclusion common in early adolescence may explain why anxiety becomes strongly linked to broader distress during this specific developmental window [57], as anxiety is often related to social stressors [58, 59]. In the Chinese educational context, the transition to middle also entails intensified academic pressure as students begin preparing for the high school entrance examination. Students in this critical period could experience a shift from implicit academic competition in primary school to a clear score‐oriented evaluation system in middle school, with the emergence of explicit class ranking and score‐based class allocation [60]. This environmental shift occurs simultaneously when adolescents are biologically the most vulnerable. While the limbic system undergoes rapid development during this period, heightening emotional reactivity, the prefrontal cortex (PFC) remains functionally immature compared to high school students [24, 25]. This developmental mismatch limits the middle‐school student’s capacity for objective stress analysis and emotional regulation.

Apart from the general patterns of comorbid risk, our analysis revealed marked sex differences in the developmental trajectories of comorbidity. Our results aligned with previous evidence showing females as consistently demonstrating higher rates of comorbid depression and anxiety compared to males [5, 28]. More importantly, the divergence in risk was not static, but followed distinct developmental patterns. While baseline risks in early adolescence (primary‐school period) were relatively comparable on harmonised scale, the disparity widened significantly following the transition to middle school, with females exhibiting a steeper acceleration in symptom severity and comorbidity risk from mid‐adolescence, especially in symptomatic participants. This widening gap aligns with the gender gap often observed in depression and anxiety post‐puberty [26, 27]. This female‐specific vulnerability might reflect the convergence of multilevel factors. Biologically, the hormonal fluctuations associated with menarche and the pubertal transition may interact with stress‐response systems, such as the HPA axis, to increase susceptibility to affective dysregulation [20]. Psychosocially, female adolescents report higher exposure to interpersonal stressors and are more likely to engage in co‐rumination [61], which has been identified as a transdiagnostic risk factor for both anxiety and depression [62]. Furthermore, our results identified a female‐specific peak in anxiety‐associated comorbidity during middle school (grades 8–9). This may correspond to the combined socioenvironmental change in female adolescents. The heightened social evaluative threat experienced by female adolescents [30], coupled with societal pressures regarding body image and social performance [63], may render anxiety symptoms particularly toxic, rapidly cascading into depressive hopelessness [64].

The sex‐specific dynamic patterns in symptomatic participants also emerged for the moderating effects of emotion regulation, supporting its transdiagnostic role for depression and anxiety [33–35]. Overall, the role of cognitive reappraisal and expression suppression are more pronounced in females. More importantly, sex‐specific paradoxical patterns emerged across sex and grade in symptomatic participants, potentially explained the conflicting evidence regarding the role of cognitive reappraisal and expression suppression in depression and anxiety [39]. Consistent with prior research [36–38], cognitive reappraisal emerged as a significant moderator reducing the link between anxiety symptoms and comorbid risk in females. Nevertheless, it also appeared to amplify the link between depressive symptoms and comorbid risk in females before grade 10. This might reflect the relatively more complex emotional processing for female than male adolescents. For female adolescents, reappraising a highly distressing social event, such as peer rejection, may demand too much effort that it becomes a stressor [31, 32], promoting rumination rather than resolution [65]. The pressure to reframe negative thoughts might also create an emotional discrepancy that, when she cannot do so, deepens her sense of inadequacy and self‐blame [66, 67]. This interpretation is consistent with recent network evidence showing that self‐critical concerns, especially the fear of disappointing others and ideal‐self discrepancy, occupy central and bridge positions in adolescent anxiety–depression comorbidity networks [68]. For younger female adolescents, attempts at cognitive reappraisal may therefore become entangled with self‐critical evaluation rather than reducing distress. This paradoxical role tend to diminish with grade, as the significant difference in the associations between depression and comorbid risk between female adolescents with high and low cognitive reappraisal scores disappeared in grades 10–12. This might be attributed to the neurodevelopmental maturation of the PFC. Cognitive reappraisal is a cognitively demanding strategy that relies heavily on top‐down control mechanisms housed in the PFC to modulate amygdala reactivity [69, 70]. In early adolescence, the limbic system is highly active, while the PFC is still undergoing maturation [24, 25]. While younger adolescents attempting to use reappraisal may lack the neural efficiency to execute it successfully, older adolescents with more matured structural connectivity between the PFC and the amygdala strengthens might be more likely to successfully execute reappraisal strategies.

A sex‐specific comorbid risk‐amplifying role of expression suppression related to anxiety symptoms also emerged for symptomatic female adolescents, as female adolescents with low expression suppression reported significantly lower anxiety‐associated comorbid risk across grades. Interestingly, the middle‐school peak for the association between anxiety and comorbid risk for females only emerged for those with high expression suppression. This indicated a potential explanatory role of expression suppression for this female‐specific critical period for comorbidity. Aligning with previous evidence showing expressive suppression being a mediator linking anxiety and depression [71], female adolescents might be more likely to suppress their feeling of anxiety to maintain peer status [72, 73]. This tendency might have induced the comorbid risk for middle‐school female adolescents. In symptomatic male adolescents, a paradoxical pattern emerged, as male adolescents with high expression suppression reported lower comorbid risk associated with depression in late adolescence (grades 11–12). While expression suppression is predominantly acknowledged as maladaptive [36–38], our results align with other evidence showing a negative association between expression suppression and mental health symptoms [74, 75]. While expression suppression might align with social expectation of masculinity, potentially helping navigating social environments [76], suppression also related to a lack of explicit emotional awareness, which could also potentially explain milder reported symptoms [77].

While existing targeted approaches against comorbidity remain focused on risk factors and symptom severity [7–9], our results advocates for a more precise and personalised prevention approach, with consideration of sex and adolescent stage. School‐based prevention programmes may benefit from prioritising anxiety management during the primary‐to‐middle school transition, when anxiety‐associated comorbid risk peaks and placing greater emphasis on depressive symptoms in late adolescence, when depression‐associated risk becomes increasingly prominent. More importantly, intervention targets should be carefully tailored to meet the features of specific developmental stages and sexes. Targeting expression suppression in female adolescents might be an efficient approach in preventing anxiety‐related comorbid risk, especially during the critical transition from primary to middle school. Conversely, cognitive reappraisal may be more useful when tailored to adolescents’ symptom profiles and developmental readiness. For younger adolescents with depressive symptoms, reappraisal‐based strategies may need to be delivered carefully, with additional support for rumination, self‐blame and social‐evaluative concerns.

Few limitations should be acknowledged when interpreting the results. The cross‐sectional design of our study limited our ability to only explore concurrent comorbidity, but not sequential comorbidity, where the two disorders do not occur at the same time. This also precluded causal explanation from our results. Moreover, this cross‐sectional data limited our ability to follow the trajectory of symptom development overtime. Another point to consider is that comorbidity‐related analyses were conducted in symptomatic participants reporting at least one clinically significant symptoms to avoid potential reflection problems [53]. Although similar risk patterns were identified in sensitivity symptom‐level analysis in full participants, the moderating role of emotion regulation identified in our study might not be applicable to adolescents without clinically significant symptoms. Moreover, the exposure variables were rescaled to a harmonised scale to ensure meaningful comparison for binary comorbid outcome. Therefore, the ORs in our results should be interpreted on a harmonised scale, which does not reflect identical unit change on the measurement instrument scales. The interpretation of grade‐varying and sex‐specific contrasts are unaffected by rescaling. A further limitation concerns unmeasured biopsychosocial confounders. Although the grade‐based time‐varying effect models adjusted for sex and perceived socioeconomic status, we did not measure other relevant biopsychosocial factors, such as parenting style, attachment security, executive functioning and academic pressure. These factors are particularly salient in the Chinese educational context and may vary substantially across grades, families and schools. Finally, although the measurement scales in this study demonstrated adequate internal consistency and measurement invariance across grades, they were not specifically developed for children and adolescents. Future longitudinal cohort studies are needed to determine whether the grade‐varying patterns identified here reflect within‐person developmental changes and to distinguish concurrent from sequential comorbidity. Future studies could also incorporate repeated symptom‐level assessments to test whether specific symptoms or processes, such as social‐evaluative anxiety, guilt, sleep disturbance, self‐criticism, or rumination, function as bridge mechanisms linking anxiety and depression across adolescence. Moreover, future research could further validate the present findings using youth‐specific instruments that provide developmentally sensitive assessments of depressive symptoms, anxiety symptoms and emotion regulation. Future research should also examine broader developmental and contextual factors, including pubertal timing, parenting style, attachment security, executive functioning, peer relationships and academic pressure, to clarify why comorbid risk peaks during specific educational transitions. Intervention studies might also be conducted to test whether stage‐ and sex‐specific prevention strategies, such as anxiety‐focused support during the primary‐to‐middle school transition or suppression‐reduction programmes for middle‐school girls, are more effective than uniform school‐based approaches.

## 5. Conclusion

Utilising TVEM with a large‐scale dataset, the current study identified dynamic patterns of risk of comorbidity between depression and anxiety across sex and adolescence stage. While supporting an overall increasing trend of comorbid risk from early to late adolescence, our results further indicated a critical transition from primary to middle school as anxiety‐related comorbid risk sharply increased, especially for adolescent girls. We also identified a more pronounced depression‐related comorbid risk in late adolescence. Sex and adolescence stage‐specific moderating roles of emotion regulation strategies emerged for comorbid risk associated with anxiety and depressive symptoms. Our results advocates for a more precise and personalised prevention approach against comorbidity, prioritising the critical transition period, and choosing intervention targets carefully with considerations of sex and adolescence stages.

## Author Contributions

Dongyu Liu and Bao Zhang conceived and designed the study. Bao Zhang carried out the study. Dongyu Liu analysed the data and wrote the original manuscript. Luxia Jia and Bao Zhang obtained funding and supervised the study.

## Funding

This study was supported by the “Research and Practice Project on Promoting High‐Quality Development of Basic Education through ’New Normal Education’ Construction” (Ref. 57) for Bao Zhang, the Guangdong Provincial Philosophy and Social Sciences Planning Project (Grant GD25YJY28) for Luxia Jia, and the Guangzhou Social Science Youth Project for Higher Education Institutions (Grant 2024312389) for Luxia Jia.

## Disclosure

All authors interpreted the data, critically edited the manuscript, and approved the final version. After using the tool, ChatGPT 5, the authors reviewed and edited the content as needed and take full responsibility for the content of the publication.

## Conflicts of Interest

The authors declare no conflicts of interest.

## Supporting Information

Additional supporting information can be found online in the Supporting Information section.

## Supporting information


**Supporting Information** All supporting information is included in a single file titled “Supplementary Document.docx”. Figure S1: Prevalence of depression and anxiety comorbidity across educational stages in (A) females and (B) males. Figure S2: Time‐varying association between depressive and anxiety symptoms. Figure S3: Combined symptoms severity associated with depressive and anxiety symptoms in (A) overall participants and (B, C) by sex. Figure S4: Risk of comorbidity associated emotion regulation. Table S1: STROBE checklist. Table S2: Measurement invariance tests for variables of interest. Table S3: Descriptive statistics. Table S4: Parametric coefficient of the association between anxiety and depressive symptoms at each grade. Table S5: Parametric coefficient of the risk of comorbidity associated with anxiety and depressive symptoms at each grade by sex. Table S6: Parametric coefficient of association between combined symptom score with individual symptoms of anxiety and depression at each grade. Table S7: Parametric coefficient of association between combined symptom score with individual symptoms of anxiety and depression at each grade by sex. Table S8: Parametric coefficients of the risk of comorbidity associated with cognitive reappraisal and expression suppression at each grade. Table S9: Parametric coefficients of the risk of comorbidity associated the interaction of cognitive reappraisal and expression suppression with anxiety and depressive symptoms at each grade by sex.

## Data Availability

The data that support the findings of this study are available from the corresponding author upon reasonable request.
