# Supplementary material for: Time‐Variant Patterns of Comorbid Depression and Anxiety Across Early to Late Adolescence: The Sex‐Specific Role of Emotion Regulation
Source: Depress Anxiety. 2026 Oct 29;2026:7875846. doi: 10.1155/da/7875846 (PMC13717479; doi:10.1155/da/7875846)
Supplement: Supplementary file 1 [file DA-2026-7875846-s001.docx]

**Time-Variant Patterns of Comorbid Depression and Anxiety Across Early to Late Adolescence: The Sex-Specific Role of Emotion Regulation**

**Supplementary Document**

Table of Contents

[Figure S1. Prevalence of depression and anxiety comorbidity across educational stages in (A) Females and (B) Males	2](#_Toc235354519)

[Figure S2. Time-varying association between depressive and anxiety symptoms	3](#_Toc235354520)

[Figure S3. Combined symptoms severity associated with depressive and anxiety symptoms in (A) overall participants and (B; C) by sex	4](#_Toc235354521)

[Figure S4. Risk of comorbidity associated emotion regulation	5](#_Toc235354522)

[Table S1. STROBE Checklist	6](#_Toc235354523)

[Table S2. Measurement invariance tests for variables of interest	8](#_Toc235354524)

[Table S3. Descriptive statistics	9](#_Toc235354525)

[Table S4. Parametric coefficient of the association between anxiety and depressive symptoms at each grade.	10](#_Toc235354526)

[Table S5. Parametric coefficient of the risk of comorbidity associated with anxiety and depressive symptoms at each grade by sex	11](#_Toc235354527)

[Table S6. Parametric coefficient of association between combined symptom score with individual symptoms of anxiety and depression at each grade.	12](#_Toc235354528)

[Table S7. Parametric coefficient of association between combined symptom score with individual symptoms of anxiety and depression at each grade by sex	13](#_Toc235354529)

[Table S8. Parametric coefficients of the risk of comorbidity associated with cognitive reappraisal and expression suppression at each grade.	14](#_Toc235354530)

[Table S9. Parametric coefficients of the risk of comorbidity associated the interaction of Cognitive Reappraisal and Expression Suppression with anxiety and depressive symptoms at each grade by sex.	15](#_Toc235354531)

# ***Figure S1.*** Prevalence of depression and anxiety comorbidity across educational stages in (A) Females and (B) Males

# ***Figure S2.*** Time-varying association between depressive and anxiety symptoms

**Note:** Standardised β coefficients are reported for anxiety symptoms predicting depression; Sex and socioeconomic status were adjusted as covariates.

# ***Figure S3.*** Combined symptoms severity associated with depressive and anxiety symptoms in (A) overall participants and (B; C) by sex

**Note:** In all participants (n = 58,622); Sex and perceived socioeconomic status were adjusted as fixed-effect covariates in (A) overall participants; Perceived socioeconomic status was adjusted as fixed effect covariate in (B; C) sex-stratified models.

# ***Figure S4.*** Risk of comorbidity associated emotion regulation

**Note:** In participants with either or both clinically significant depression and anxiety (n = 20,473). Comorbidity was defined as having both clinically significant depression and anxiety. Sex and perceived socioeconomic status were adjusted as fixed-effect covariates

| **Table S1.** STROBE Checklist | | | |
| --- | --- | --- | --- |
|  | **Item No.** | **Recommendation** | **Appeared in manuscript** |
| **Title and abstract** | 1 | (*a*) Indicate the study’s design with a commonly used term in the title or the abstract | Title Page |
|  |  | (*b*) Provide in the abstract an informative and balanced summary of what was done and what was found | Abstract section |
| **Introduction** | | |  |
| Background/rationale | 2 | Explain the scientific background and rationale for the investigation being reported | Introduction, Paragraph 1-3 |
| Objectives | 3 | State specific objectives, including any prespecified hypotheses | Introduction Paragraph 4 |
| **Methods** | | |  |
| Study design | 4 | Present key elements of study design early in the paper | Methods, Procedure and Participants section |
| Setting | 5 | Describe the setting, locations, and relevant dates, including periods of recruitment, exposure, follow-up, and data collection | Methods, Procedure and Participants section |
| Participants | 6 | (*a*) Give the eligibility criteria, and the sources and methods of selection of participants | Methods, Procedure and Participants section |
| Variables | 7 | Clearly define all outcomes, exposures, predictors, potential confounders, and effect modifiers. Give diagnostic criteria, if applicable | Methods, Measurement section |
| Data sources/ measurement | 8 | For each variable of interest, give sources of data and details of methods of assessment (measurement). Describe comparability of assessment methods if there is more than one group | Methods, Procedure and Participants section and Measurement section |
| Bias | 9 | Describe any efforts to address potential sources of bias | Methods, Covariates section, Statistics section Paragraph 3-4 |
| Study size | 10 | Explain how the study size was arrived at | Methods, Procedure and Participants section |
| Quantitative variables | 11 | Explain how quantitative variables were handled in the analyses. If applicable, describe which groupings were chosen and why | Methods, Statistics section Paragraph 1 |
| Statistical methods | 12 | (*a*) Describe all statistical methods, including those used to control for confounding | Methods, Statistics section Paragraph 2-4 |
|  |  | (*b*) Describe any methods used to examine subgroups and interactions | Methods, Statistics section Paragraph 5 |
|  |  | (*c*) Explain how missing data were addressed | Methods, Procedure and Participants section |
|  |  | (*d*) If applicable, describe analytical methods taking account of sampling strategy | NA |
|  |  | (*e*) Describe any sensitivity analyses | NA |
| **Results** | | |  |
| Participants | 13 | (a) Report numbers of individuals at each stage of study—eg numbers potentially eligible, examined for eligibility, confirmed eligible, included in the study, completing follow-up, and analysed | Methods, Procedure and Participants section; Results, Paragraph 1 |
|  |  | (b) Give reasons for non-participation at each stage | Methods, Procedure and Participants section |
|  |  | (c) Consider use of a flow diagram | NA |
| Descriptive data | 14 | (a) Give characteristics of study participants (eg demographic, clinical, social) and information on exposures and potential confounders | Results, Paragraph 1 |
|  |  | (b) Indicate number of participants with missing data for each variable of interest | Methods, Procedure and Participants section |
| Outcome data | 15 | Report numbers of outcome events or summary measures | Supplementary Table S3 |
| Main results | 16 | (*a*) Give unadjusted estimates and, if applicable, confounder-adjusted estimates and their precision (eg, 95% confidence interval). Make clear which confounders were adjusted for and why they were included | Results, Comorbidity Between Depression and Anxiety section |
|  |  | (*b*) Report category boundaries when continuous variables were categorized | Methods, Measurement section |
|  |  | (*c*) If relevant, consider translating estimates of relative risk into absolute risk for a meaningful time period | NA |
| Other analyses | 17 | Report other analyses done—eg analyses of subgroups and interactions, and sensitivity analyses | Results, Comorbidity Risk by Sex section; Sex-specific Moderating Role of Emotion Regulation section |
| **Discussion** | | |  |
| Key results | 18 | Summarise key results with reference to study objectives | Discussion, Paragraph 1 |
| Limitations | 19 | Discuss limitations of the study, taking into account sources of potential bias or imprecision. Discuss both direction and magnitude of any potential bias | Discussion, Paragraph 7 |
| Interpretation | 20 | Give a cautious overall interpretation of results considering objectives, limitations, multiplicity of analyses, results from similar studies, and other relevant evidence | Discussion, Paragraph 2-6 |
| Generalisability | 21 | Discuss the generalisability (external validity) of the study results | Discussion, Paragraph 6-7 |
| **Other information** | | |  |
| Funding | 22 | Give the source of funding and the role of the funders for the present study and, if applicable, for the original study on which the present article is based | Funding statement section |

| **Table S2.** Measurement invariance tests for variables of interest | | | | | | | | | | | |
| --- | --- | --- | --- | --- | --- | --- | --- | --- | --- | --- | --- |
| **Construct** | **Model** | **χ²** | **df** | **CFI** | **TLI** | **RMSEA** | **SRMR** | **ΔCFI** | **ΔRMSEA** | **ΔSRMR** | **Invariance** **^†^** |
| PHQ-9 Depression | Configural | 8092.76 | 243 | 0.9872 | 0.9829 | 0.0702 | 0.0284 |  |  |  |  |
|  | Metric | 5336.05 | 307 | 0.9918 | 0.9913 | 0.0500 | 0.0322 | 0.0046 | -0.0202 | 0.0037 | Supported |
|  | Scalar | 7116.58 | 379 | 0.9890 | 0.9906 | 0.0521 | 0.0288 | -0.0028 | 0.0021 | -0.0033 | Supported |
|  | Strict | 10928.46 | 451 | 0.9829 | 0.9877 | 0.0595 | 0.0337 | -0.0061 | 0.0075 | 0.0049 | Supported |
| GAD-7 Anxiety | Configural | 10555.78 | 126 | 0.9890 | 0.9835 | 0.1124 | 0.0276 |  |  |  |  |
|  | Metric | 6266.64 | 174 | 0.9936 | 0.9930 | 0.0731 | 0.0286 | 0.0046 | -0.0393 | 0.0010 | Supported |
|  | Scalar | 8714.02 | 230 | 0.9911 | 0.9927 | 0.0750 | 0.0277 | -0.0025 | 0.0019 | -0.0009 | Supported |
|  | Strict | 14608.40 | 286 | 0.9849 | 0.9900 | 0.0874 | 0.0316 | -0.0061 | 0.0124 | 0.0039 | Supported |
| Cognitive Reappraisal | Configural | 13189.39 | 81 | 0.9860 | 0.9767 | 0.1571 | 0.0364 |  |  |  |  |
|  | Metric | 7071.94 | 121 | 0.9926 | 0.9917 | 0.0936 | 0.0409 | 0.0066 | -0.0635 | 0.0044 | Supported |
|  | Scalar | 9353.22 | 217 | 0.9903 | 0.9939 | 0.0802 | 0.0368 | -0.0023 | -0.0135 | -0.0041 | Supported |
|  | Strict | 15064.26 | 265 | 0.9842 | 0.9920 | 0.0923 | 0.0415 | -0.0060 | 0.0122 | 0.0047 | Supported |
| Expression Suppression | Configural | 531.39 | 18 | 0.9971 | 0.9914 | 0.0660 | 0.0131 |  |  |  |  |
|  | Metric | 681.04 | 42 | 0.9964 | 0.9954 | 0.0482 | 0.0177 | -0.0007 | -0.0178 | 0.0046 | Supported |
|  | Scalar | 1025.86 | 106 | 0.9949 | 0.9974 | 0.0364 | 0.0150 | -0.0016 | -0.0118 | -0.0027 | Supported |
|  | Strict | 8236.56 | 138 | 0.9547 | 0.9823 | 0.0946 | 0.0295 | -0.0402 | 0.0582 | 0.0145 | Not supported |

**Note:** Single-factor model was applied. PHQ-9 = 9-item patient health questionnaire; GAD-7 = 7-item generalised anxiety disorder questionnaire; **†** Metric invariance was supported if ΔCFI ≤ 0.010 & ΔRMSEA ≤ 0.015 & ΔSRMR ≤ 0.030, scalar and strict invariances were supported if ΔCFI ≤ 0.010 & ΔRMSEA ≤ 0.015 & ΔSRMR ≤ 0.010

# **Table S3.** Descriptive statistics

|  | **Overall (N=58622)** |
| --- | --- |
| **Sex** |  |
| Female | 26884 (45.9%) |
| Male | 31738 (54.1%) |
| **Subjective Socio-Economic Status** |  |
| Mean (SD) | 5.13 (1.61) |
| Median [Min, Max] | 5.00 [1.00, 10.0] |
| **Depressive Symptoms** |  |
| Mean (SD) | 8.28 (5.92) |
| Median [Min, Max] | 8.01 [0, 27.0] |
| **Clinically Significant Depression** |  |
| Yes | 16495 (28.1%) |
| **Anxiety Symptoms** |  |
| Mean (SD) | 6.30 (5.20) |
| Median [Min, Max] | 6.02 [0, 21.0] |
| **Clinically Significant Anxiety** |  |
| Yes | 15613 (26.6%) |
| **Depression and Anxiety Comorbidity** |  |
| Yes | 11806 (20.1%) |
| **Cognitive Reappraisal** |  |
| Mean (SD) | 21.9 (6.57) |
| Median [Min, Max] | 23.0 [0, 30.0] |
| **Expression Suppression** |  |
| Mean (SD) | 11.1 (4.48) |
| Median [Min, Max] | 11.0 [0, 20.0] |

**Note:** Comorbidity was defined as having both clinically significant depression and anxiety.

# **Table S4.** Parametric coefficient of the association between anxiety and depressive symptoms at each grade.

| **Grade (Age IQR)** | **Parametric Coefficient β [95% CI]** |
| --- | --- |
| G4 (10-11) | 0.80 [0.75, 0.85] |
| G5 (11-12) | 0.81 [0.77, 0.85] |
| G6 (11-13) | 0.83 [0.79, 0.87] |
| G7 (12-14) | 0.83 [0.79, 0.87] |
| G8 (13-14) | 0.83 [0.78, 0.87] |
| G9 (14-15) | 0.82 [0.77, 0.86] |
| G10 (15-16) | 0.80 [0.76, 0.84] |
| G11 (16-17) | 0.78 [0.73, 0.82] |
| G12 (17-18) | 0.76 [0.71, 0.82] |

**Note:** IQR = interquartile range; Standardised β coefficients are reported for anxiety symptoms predicting depression; Sex and perceived socioeconomic status were adjusted as fixed-effect covariates.

| **Table S5.** Parametric coefficient of the risk of comorbidity associated with anxiety and depressive symptoms at each grade by sex | | | | | | |
| --- | --- | --- | --- | --- | --- | --- |
| **Grade**  (Age IQR) | **Parametric Coefficient OR [95% CI]** | | | | | |
|  | **Anxiety** | | | **Depression** | | |
|  | **Female** | **Male** | **Ratio** | **Female** | **Male** | **Ratio** |
| G4 (10-11) | 1.66 [1.60, 1.73] | 1.53 [1.45, 1.62] | 0.92 [0.87, 0.99] | 1.55 [1.33, 1.81] | 1.47 [1.35, 1.61] | 0.95 [0.82, 1.00] |
| G5 (11-12) | 1.68 [1.63, 1.73] | 1.55 [1.47, 1.63] | 0.92 [0.88, 0.98] | 1.61 [1.38, 1.87] | 1.50 [1.38, 1.63] | 0.93 [0.86, 0.98] |
| G6 (11-13) | 1.71 [1.66, 1.76] | 1.57 [1.49, 1.65] | 0.92 [0.89, 0.97] | 1.67 [1.44, 1.93] | 1.53 [1.41, 1.66] | 0.92 [0.88, 0.98] |
| G7 (12-14) | 1.73 [1.68, 1.79] | 1.58 [1.51, 1.66] | 0.92 [0.89, 0.96] | 1.73 [1.49, 2.00] | 1.56 [1.43, 1.69] | 0.90 [0.88, 0.97] |
| G8 (13-14) | 1.74 [1.69, 1.80] | 1.60 [1.52, 1.68] | 0.92 [0.89, 0.96] | 1.79 [1.55, 2.07] | 1.58 [1.46, 1.72] | 0.89 [0.86, 0.94] |
| G9 (14-15) | 1.74 [1.69, 1.80] | 1.62 [1.54, 1.70] | 0.93 [0.90, 0.97] | 1.85 [1.60, 2.14] | 1.61 [1.49, 1.75] | 0.87 [0.84, 0.92] |
| G10 (15-16) | 1.73 [1.68, 1.79] | 1.64 [1.56, 1.72] | 0.94 [0.91, 0.99] | 1.92 [1.65, 2.22] | 1.64 [1.51, 1.79] | 0.86 [0.81, 0.91] |
| G11 (16-17) | 1.72 [1.67, 1.78] | 1.66 [1.57, 1.75] | 0.96 [0.92, 1.02] | 1.99 [1.71, 2.31] | 1.68 [1.54, 1.82] | 0.84 [0.78, 0.90] |
| G12 (17-18) | 1.71 [1.64, 1.78] | 1.67 [1.58, 1.77] | 0.98 [0.93, 1.07] | 2.06 [1.77, 2.39] | 1.71 [1.56, 1.86] | 0.83 [0.74, 0.90] |

**Note:** In participants with either or both clinically significant depression and anxiety (n = 20,473); Comorbidity was defined as having both clinically significant depression and anxiety; IOR=Interquartile range; Ratio indicates the difference between ORs in females and males at each grade, with Rato > 1 indicating higher OR in males than males. Perceived socioeconomic status was adjusted as fixed effect covariate.

| **Table S6.** Parametric coefficient of association between combined symptom score with individual symptoms of anxiety and depression at each grade. | | | | | | | | | |
| --- | --- | --- | --- | --- | --- | --- | --- | --- | --- |
| **Grade**  (Age IQR) | **Parametric Coefficient β [95% CI]** | | | | | | | | |
|  | **Overall Sample** | | | **Females** | | | **Males** | | |
|  | **Anxiety** | **Depression** | **Difference** | **Anxiety** | **Depression** | **Difference** | **Anxiety** | **Depression** | **Difference** |
| G4 (10-11) | 1.80 [1.75, 1.85] | 1.78 [1.74, 1.82] | 0.02 [-0.01, 0.03] | 1.82 [1.75, 1.89] | 1.81 [1.75, 1.86] | 0.01 [-0.02, 0.04] | 1.78 [1.73, 1.83] | 1.77 [1.73, 1.81] | 0.01 [-0.02, 0.04] |
| G5 (11-12) | 1.81 [1.77, 1.85] | 1.79 [1.75, 1.83] | 0.02 [0.00, 0.03] | 1.83 [1.78, 1.89] | 1.81 [1.76, 1.86] | 0.02 [-0.00, 0.04] | 1.79 [1.75, 1.84] | 1.78 [1.74, 1.82] | 0.02 [-0.01, 0.04] |
| G6 (11-13) | 1.83 [1.79, 1.87] | 1.80 [1.76, 1.84] | 0.03 [0.02, 0.05] | 1.84 [1.79, 1.90] | 1.81 [1.77, 1.86] | 0.03 [0.01, 0.04] | 1.81 [1.77, 1.85] | 1.79 [1.75, 1.83] | 0.02 [-0.00, 0.04] |
| G7 (12-14) | 1.83 [1.79, 1.87] | 1.81 [1.77, 1.85] | 0.02 [0.01, 0.03] | 1.85 [1.79, 1.91] | 1.82 [1.78, 1.87] | 0.03 [0.01, 0.04] | 1.81 [1.77, 1.85] | 1.80 [1.76, 1.84] | 0.01 [-0.01, 0.02] |
| G8 (13-14) | 1.83 [1.78, 1.87] | 1.82 [1.78, 1.86] | 0.01 [0.00, 0.02] | 1.85 [1.79, 1.91] | 1.83 [1.79, 1.87] | 0.02 [0.00, 0.04] | 1.81 [1.76, 1.85] | 1.81 [1.77, 1.85] | 0.00 [-0.03, 0.01] |
| G9 (14-15) | 1.82 [1.77, 1.86] | 1.83 [1.79, 1.87] | -0.01 [-0.02, 0.01] | 1.83 [1.77, 1.89] | 1.84 [1.80, 1.89] | -0.01 [-0.02, 0.01] | 1.81 [1.76, 1.85] | 1.82 [1.78, 1.86] | -0.01 [-0.03, 0.01] |
| G10 (15-16) | 1.80 [1.76, 1.84] | 1.84 [1.80, 1.88] | -0.04 [-0.07, -0.03] | 1.80 [1.75, 1.86] | 1.85 [1.81, 1.90] | -0.05 [-0.08, -0.04] | 1.8 [1.76, 1.84] | 1.82 [1.78, 1.87] | -0.03 [-0.05, -0.01] |
| G11 (16-17) | 1.78 [1.73, 1.82] | 1.85 [1.81, 1.89] | -0.07 [-0.09, -0.06] | 1.78 [1.72, 1.83] | 1.87 [1.82, 1.91] | -0.09 [-0.11, -0.06] | 1.79 [1.74, 1.83] | 1.83 [1.79, 1.88] | -0.04 [-0.06, -0.02] |
| G12 (17-18) | 1.76 [1.71, 1.82] | 1.86 [1.82, 1.90] | -0.09 [-0.11, -0.05] | 1.76 [1.69, 1.84] | 1.88 [1.82, 1.93] | -0.12 [-0.14, -0.06] | 1.78 [1.72, 1.83] | 1.84 [1.80, 1.88] | -0.07 [-0.09, -0.02] |

**Note:** In all participants (n = 58,622); IOR=Interquartile range; β difference = The difference between anxiety and depression-related β at each grade, with Difference > 0 indicating higher Anxiety-related than depression related β. Sex and perceived socioeconomic status were adjusted as fixed-effect covariates in overall participants; Perceived socioeconomic status was adjusted as fixed effect covariate in sex-stratified models.

| **Table S7.** Parametric coefficient of association between combined symptom score with individual symptoms of anxiety and depression at each grade by sex | | | | | | |
| --- | --- | --- | --- | --- | --- | --- |
| **Grade**  (Age IQR) | **Parametric Coefficient β [95% CI]** | | | | | |
|  | **Anxiety** | | | **Depression** | | |
|  | **Female** | **Male** | **Difference** | **Female** | **Male** | **Difference** |
| G4 (10-11) | 1.82 [1.75, 1.89] | 1.78 [1.73, 1.83] | -0.04 [-0.07, -0.01] | 1.81 [1.75, 1.86] | 1.77 [1.73, 1.81] | -0.04 [-0.07, -0.01] |
| G5 (11-12) | 1.83 [1.78, 1.89] | 1.79 [1.75, 1.84] | -0.04 [-0.06, -0.02] | 1.81 [1.76, 1.86] | 1.78 [1.74, 1.82] | -0.03 [-0.05, -0.01] |
| G6 (11-13) | 1.84 [1.79, 1.90] | 1.81 [1.77, 1.85] | -0.03 [-0.05, -0.00] | 1.81 [1.77, 1.86] | 1.79 [1.75, 1.83] | -0.03 [-0.04, -0.01] |
| G7 (12-14) | 1.85 [1.79, 1.91] | 1.81 [1.77, 1.85] | -0.04 [-0.05, -0.02] | 1.82 [1.78, 1.87] | 1.80 [1.76, 1.84] | -0.02 [-0.03, -0.01] |
| G8 (13-14) | 1.85 [1.79, 1.91] | 1.81 [1.76, 1.85] | -0.04 [-0.07, -0.03] | 1.83 [1.79, 1.87] | 1.81 [1.77, 1.85] | -0.02 [-0.03, -0.01] |
| G9 (14-15) | 1.83 [1.77, 1.89] | 1.81 [1.76, 1.85] | -0.03 [-0.04, -0.00] | 1.84 [1.80, 1.89] | 1.82 [1.78, 1.86] | -0.02 [-0.04, -0.01] |
| G10 (15-16) | 1.80 [1.75, 1.86] | 1.80 [1.76, 1.84] | 0.00 [-0.03, 0.02] | 1.85 [1.81, 1.90] | 1.82 [1.78, 1.87] | -0.03 [-0.04, -0.01] |
| G11 (16-17) | 1.78 [1.72, 1.83] | 1.79 [1.74, 1.83] | 0.01 [-0.02, 0.04] | 1.87 [1.82, 1.91] | 1.83 [1.79, 1.88] | -0.03 [-0.06, -0.01] |
| G12 (17-18) | 1.76 [1.69, 1.84] | 1.78 [1.72, 1.83] | 0.01 [-0.04, 0.05] | 1.88 [1.82, 1.93] | 1.84 [1.80, 1.88] | -0.04 [-0.08, -0.01] |

**Note:** In all participants (n = 58,622); IOR=Interquartile range; β difference = The difference between effect sizes in females and males at each grade, with Difference > 0 indicating higher β in males. Perceived socioeconomic status was adjusted as fixed effect covariate in sex-stratified models

# **Table S8.** Parametric coefficients of the risk of comorbidity associated with cognitive reappraisal and expression suppression at each grade.

| **Grade**  (Age IQR) | **Parametric Coefficient OR [95% CI]** | |
| --- | --- | --- |
|  | **Cognitive Reappraisal** | **Expression Suppression** |
| G4 (10-11) | 0.96 [0.94, 0.99] | 1.04 [1.04, 1.04] |
| G5 (11-12) | 0.96 [0.94, 0.98] | 1.04 [1.04, 1.04] |
| G6 (11-13) | 0.95 [0.93, 0.98] | 1.04 [1.04, 1.04] |
| G7 (12-14) | 0.95 [0.93, 0.97] | 1.04 [1.04, 1.04] |
| G8 (13-14) | 0.94 [0.92, 0.97] | 1.04 [1.04, 1.04] |
| G9 (14-15) | 0.94 [0.92, 0.96] | 1.04 [1.04, 1.04] |
| G10 (15-16) | 0.94 [0.91, 0.96] | 1.04 [1.04, 1.04] |
| G11 (16-17) | 0.93 [0.91, 0.95] | 1.04 [1.04, 1.04] |
| G12 (17-18) | 0.93 [0.90, 0.95] | 1.04 [1.04, 1.04] |

**Note:** In participants with either or both clinically significant depression and anxiety (n = 20,473). Comorbidity was defined as having both clinically significant depression and anxiety. Sex and perceived socioeconomic status were adjusted as fixed-effect covariates

# **Table S9.** Parametric coefficients of the risk of comorbidity associated the interaction of Cognitive Reappraisal and Expression Suppression with anxiety and depressive symptoms at each grade by sex.

| **Grade**  (Age IQR) | **Parametric Coefficient OR [95% CI]** | | | | |
| --- | --- | --- | --- | --- | --- |
|  | **Females** | |  | **Males** | |
|  | **Anxiety symptoms** | **Depressive symptoms** |  | **Anxiety symptoms** | **Depressive symptoms** |
| **Interaction with Cognitive Reappraisal** | | | | | |
| G4 (10-11) | 0.994 [0.994, 0.994] | 1.007 [1.007, 1.007] |  | 1.000 [1.000, 1.000] | 1.001 [1.000, 1.001] |
| G5 (11-12) | 0.994 [0.994, 0.994] | 1.007 [1.007, 1.007] |  | 1.000 [1.000, 1.000] | 1.001 [1.000, 1.001] |
| G6 (11-13) | 0.994 [0.994, 0.994] | 1.007 [1.007, 1.007] |  | 1.000 [1.000, 1.000] | 1.001 [1.000, 1.001] |
| G7 (12-14) | 0.994 [0.994, 0.994] | 1.007 [1.007, 1.007] |  | 1.000 [1.000, 1.000] | 1.001 [1.000, 1.001] |
| G8 (13-14) | 0.994 [0.994, 0.994] | 1.007 [1.007, 1.007] |  | 1.000 [1.000, 1.000] | 1.001 [1.000, 1.001] |
| G9 (14-15) | 0.994 [0.994, 0.994] | 1.007 [1.007, 1.007] |  | 1.000 [1.000, 1.000] | 1.001 [1.000, 1.001] |
| G10 (15-16) | 0.994 [0.994, 0.994] | 1.007 [1.007, 1.007] |  | 1.000 [1.000, 1.000] | 1.001 [1.000, 1.001] |
| G11 (16-17) | 0.994 [0.994, 0.994] | 1.007 [1.007, 1.007] |  | 1.000 [1.000, 1.000] | 1.001 [1.000, 1.001] |
| G12 (17-18) | 0.994 [0.994, 0.994] | 1.007 [1.007, 1.007] |  | 1.000 [1.000, 1.000] | 1.001 [1.000, 1.001] |
| **Interaction with Expression Suppression** | | | | | |
| G4 (10-11) | 1.008 [1.005, 1.011] | 0.998 [0.997, 1.000] |  | 1.005 [1.005, 1.005] | 0.999 [0.996, 1.003] |
| G5 (11-12) | 1.009 [1.006, 1.011] | 0.998 [0.997, 1.000] |  | 1.005 [1.005, 1.005] | 1.000 [0.997, 1.003] |
| G6 (11-13) | 1.009 [1.007, 1.012] | 0.998 [0.997, 1.000] |  | 1.005 [1.005, 1.005] | 1.001 [0.998, 1.004] |
| G7 (12-14) | 1.009 [1.008, 1.012] | 0.998 [0.997, 1.000] |  | 1.005 [1.005, 1.005] | 1.001 [0.998, 1.004] |
| G8 (13-14) | 1.010 [1.008, 1.012] | 0.998 [0.997, 1.000] |  | 1.005 [1.005, 1.005] | 1.002 [0.999, 1.005] |
| G9 (14-15) | 1.010 [1.007, 1.012] | 0.998 [0.997, 1.000] |  | 1.005 [1.005, 1.005] | 1.003 [1.001, 1.006] |
| G10 (15-16) | 1.009 [1.007, 1.011] | 0.998 [0.997, 1.000] |  | 1.005 [1.005, 1.005] | 1.003 [1.001, 1.006] |
| G11 (16-17) | 1.008 [1.005, 1.011] | 0.998 [0.997, 1.000] |  | 1.005 [1.005, 1.005] | 1.004 [1.001, 1.007] |
| G12 (17-18) | 1.007 [1.004, 1.011] | 0.998 [0.997, 1.000] |  | 1.005 [1.005, 1.005] | 1.005 [1.001, 1.008] |

**Note:** In participants with either or both clinically significant depression and anxiety (n = 20,473). Comorbidity was defined as having both clinically significant depression and anxiety. Perceived socioeconomic status was adjusted as fixed-effect covariate.
